# Treatment Patterns of Patients With Pulmonary Hypertension: A Descriptive Study in Colombia

**DOI:** 10.1111/crj.70063

**Published:** 2025-02-20

**Authors:** Manuel Machado‐Duque, Andrés Gaviria‐Mendoza, Luis Fernando Valladales‐Restrepo, Manuel Pacheco, Juan Sebastián Franco, María del Rosario Forero, Rubiela Suarez, Oscar Peñuela, Jorge E. Machado‐Alba

**Affiliations:** ^1^ Grupo de Investigación en Farmacoepidemiología y Farmacovigilancia Universidad Tecnológica de Pereira y Audifarma S.A Pereira Colombia; ^2^ Grupo Biomedicina Fundación Universitaria Autónoma de las Américas Pereira Colombia; ^3^ Universidad Tecnológica de Pereira Pereira Colombia; ^4^ Medical Affairs, Bayer S.A. Bogotá Colombia

**Keywords:** calcium channel blockers, endothelin receptor antagonists, pulmonary hypertension, pharmacoepidemiology, phosphodiesterase 5 inhibitors

## Abstract

**Introduction:**

Pulmonary hypertension (PH) is a chronic disease characterized by a progressive rise in pulmonary artery blood pressure. The objective was to describe the treatment patterns among ambulatory patients with pulmonary arterial hypertension (PAH) and chronic thromboembolic pulmonary hypertension (CTEPH) in a real‐world setting.

**Methods:**

This is a longitudinal cohort follow‐up study characterizing the treatment patterns of patients diagnosed with PAH or CTEPH, with secondary data from a population‐based drug‐dispensing database between 2022 and 2023, which includes sociodemographic, diagnosis, prescribing specialty, and treatment (drugs, persistence of use, and concomitant medications).

**Results:**

In total, 1045 patients with a diagnosis of PH were identified, with mean age of 62.9 ± 18.2 years, and 72.3% of females; of which 947 (90.6%) received monotherapy, and 98 (9.4%) received combination therapy at the beginning of follow‐up. The most frequently used drugs for the treatment of PH were calcium channel blockers (58.1%), followed by phosphodiesterase 5 inhibitors (41.1%), endothelin receptor antagonist (32.5%), and guanylate cyclase stimulants (9.7%). The schemes used most frequently were monotherapy with amlodipine (31.0%), sildenafil (19.2%), or nifedipine (10.0%), but the main combination were sildenafil with nifedipine (2.5%). The mean of persistence of use was 161 ± 123 days during 1 year of follow‐up.

**Conclusions:**

This group of patients with PH from Colombia were treated predominantly with monotherapy of calcium channel blockers and phosphodiesterase 5 inhibitors. However, current clinical practice guidelines recommend the use of combined therapy. The average persistence of the use of drugs for treatment for less than 6 months may be associated with difficulties in follow‐up, adherence, effectiveness, tolerability, and access.

## Graphical abstract

This study describes the treatment patterns among ambulatory patients with pulmonary hypertension (PH) in Colombia, from a population‐based drug‐dispensing database between 2022 and 2023. It found that patients with PH from Colombia were treated predominantly with monotherapy of calcium channel blockers and phosphodiesterase 5 inhibitors, with an average persistence of less than 6 months.

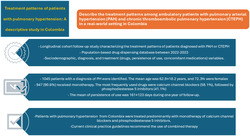

## 1 Introduction

Pulmonary hypertension (PH) is a chronic and progressive disease characterized by remodeling of the pulmonary vasculature and a progressive rise in pulmonary artery blood pressure, with a sustained and increased mean resting pulmonary artery blood pressure greater than 20 mmHg, as assessed by right heart catheterization [1, 2]. PH is an increasingly recognized comorbidity of numerous common diseases and is associated with poor prognosis. PH can be classified into five groups according to etiology. Group 1 includes primary pulmonary hypertension (PH) or pulmonary arterial hypertension (PAH). In this group, idiopathic PAH is the most common condition, with other causes, such as toxin‐induced PAH, connective tissue disorders, and other related conditions, being less prevalent. Group 2 includes PH due to left heart disease, which is the most common cause of PH. Group 3 PH is caused by lung diseases such as chronic obstructive pulmonary disease and interstitial lung disease. Group 4 PH is caused by chronic thromboembolic pulmonary hypertension (CTEPH), and Group 5 PH has uncleared or multifactorial causes [1, 3].

The reported incidence of PH in developed countries is 1.1–7.6 per million adults per year, and the prevalence is 6.6–26.0 per million adults [4]. In Colombia, according to a cross‐sectional study based on data from the National Health Ministry between 2010 and 2014, the estimated prevalence rates were approximately 63 and 39 per million people, respectively. Other local studies have reported a prevalence of 52.5 per million inhabitants, with a female predominance [5]. To date, PH continues to be a challenging, progressive and devastating chronic disease [6], and despite the current treatment options, the mortality rate remains unacceptably high. In a national registry in the United States between September 2015 and September 2020, researchers reported a mortality rate of 12.9% during a 3‐year follow‐up period. These findings suggest the importance of early diagnosis and the aggressive and appropriate use of viable therapies [7].

PAH and CTEPH are the only groups that receive specific medical treatment [1]. For the other groups, treatment is focused on the primary disease cause. Insights into the pathophysiology of PAH have led to the development of targeted treatments that improve exercise capacity, hemodynamics, and symptoms. The 2022 European Society of Cardiology (ESC)/European Respiratory Society (ERS) guidelines recommend the use of targeted therapies for PAH according to their risk assessment, with the initial combination of a phosphodiesterase‐5 inhibitor (PDE5i) and an endothelin receptor antagonist (ERA) and the addition of IV/SC prostacyclin agents in intermediate‐, high‐, or high‐risk patients (level IIa recommendation). In addition, the guidelines consider transition therapy from PDE5i to riociguat or triple therapy with prostacyclin analogs (level IIb recommendation) [2]. In the case of CTEPH, pulmonary thromboendarterectomy is the treatment of choice because it is potentially curative, whereas riociguat, the only approved and recommended medical therapy for CTEPH, is indicated for symptomatic patients with inoperable CTEPH or persistent/recurrent PH (level I recommendation) [2].

In Colombia, PH is classified as an orphan disease with a mandatory notification to the National Surveillance Program, which has allowed for an increased focus on the diagnosis and management of the affected population. Although all the pharmacological options required for the treatment of patients with PAH and CTEPH are available in Colombia, given the complexity of the treatment algorithms, it is essential to ensure that the physicians who are involved in the management of PH understand the patient profiles for the various treatment options to ensure a comprehensive clinical approach. However, information on the current treatment patterns for PH in clinical practice in Colombia is lacking. Thus, the objective of this study was to describe the treatment patterns among ambulatory patients with PAH and CTEPH that would allow us to accurately identify the treatment combinations and sequential choices most frequently used by physicians in a real‐world setting.

## 2 Materials and Methods

### 2.1 Study Design and Patients

This is a descriptive, longitudinal cohort follow‐up study with the aim of characterizing the treatment patterns of patients diagnosed with PAH or CTEPH, with secondary data from a population‐based drug‐dispensing database. The treatment patterns of prevalent patients with PAH or CTEPH from July 1, 2022, to June 30, 2023, were evaluated. Initially, all patients with an International Classification of Diseases 10 (ICD10) diagnosis related to PH (i.e., I27X) were identified in the database from January 1, 2016, to June 30, 2022. All patients with the ICD10 code I270 “Primary Pulmonary Hypertension” were classified as having PAH (Group 1 WHO). Patients with chronic thromboembolic pulmonary hypertension were obtained by combining the diagnosis I272 “Other Secondary Pulmonary Hypertension” with the codes that indicate that the patient has suffered a thromboembolic event: I260/I269 “Pulmonary Embolism” (with and without cor pulmonale), I288 “Other diseases of pulmonary vessels” and I82 “Other venous embolism and thrombosis.”

Patients aged 18 years or older with at least 1 year of enrollment with their insurance provider and at least 1 year of available data following their first recorded outpatient health contact were included. Additionally, patients with a diagnosis of PAH or CTEPH in the drug dispensing database according to the ICD‐10 codes, as described above, identified from July 1, 2022, to June 30, 2023, with a follow‐up time of 12 months, were ultimately included. Each medication that is registered in the database was effectively dispensed to the patient to whom it was delivered, and the index date was the first date of a prescription with a medication for the treatment of PAH or CTEPH documented in the database during the observation period.

### 2.2 Variables

From the medication consumption information for the population meeting the inclusion criteria, a database containing the following data was constructed:

- Sociodemographic data: sex, age, city in which the patient received care, region of Colombia, and affiliation regime.
- Diagnosis: pulmonary arterial hypertension (Group 1), chronic thromboembolic pulmonary hypertension (Group 4).
- Prescribing specialty
- Treatment patterns: Patients were prescribed medication to control PH. Data on the medication used, frequency of use, dose used, DDD (defined daily dose), range, dosing interval, and age and sex of the patients were collected. Groups: endothelin receptor antagonists (ERA); calcium channel blockers; soluble guanylate cyclase stimulator; prostacyclin receptor agonists; phosphodiesterase 5 inhibitors; and systemic and inhaled prostacyclin analogs. Grouping of combined therapies was carried out. The ratio between the mean dose and the daily dose defined by the World Health Organization was estimated.
- Treatment persistence: From the index date, the continuity of the dispensing of the medication was monitored during the 12‐month follow‐up, with continuity being considered if there was no gap in treatment > 30 days between the end of the supply and the next dispensation. The first therapy change was also identified.
- Concomitant medications: the use of additional therapies such as antihypertensives, diuretics, lipid‐lowering agents, antiulcer agents, antidiabetics, psychotropic drugs, and so on.

### 2.3 Data Analysis

The statistical analyses were explorative and descriptive in nature. The study did not aim to decide on predefined hypotheses.

The data were analyzed with the statistical package SPSS Statistics, version 28.0 for Windows (IBM, USA). A descriptive analysis with frequencies and proportions was carried out for the qualitative variables, and for all continuous variables, the mean, standard deviation, median, percentiles, and interquartile range (min, Q25, Q75 and max) are presented. The values were not imputed for variables that did not have complete information. UpSet library version‐0.6.1 in Python was used to plot the treatment patterns and combinations. Rv4.3.2 and the Sankey library were used for the Sankey plots (first vs. final treatment). This study provides real‐world data showing raw results obtained as presented in each patient's records.

### 2.4 Ethical Considerations

The protocol received endorsement from the Bioethics Committee of the Universidad in Colombia under the classification of research “without risk” (approval code: 128‐201123). According to Resolution 8430 of 1993 of the Ministry of Health of Colombia, risk‐free research does not require the signing of informed consent if it is information obtained from databases or clinical records. The research was performed with the authorization of the drug dispensing company that owns the database. The principles for the confidentiality of information established by the Declaration of Helsinki were observed.

## 3 Results

### 3.1 Sociodemographic Characteristics

In total, 1045 patients with a diagnosis of PH who met the inclusion criteria (PAH, 1027 patients; CTEPH, 18 patients) were evaluated, with a mean age of 62.9 ± 18.2 (range: 18–101) years and a predominance of females (72.3%), mainly from the Bogotá‐Cundinamarca and central regions, and were affiliated with the contributory regime. The prescriptions were made by general medicine, and the main diagnosis was primary PH (ICD10 code: I270) (see Table 1).

**TABLE 1:** Sociodemographic and clinical variables in a group of 1045 patients with pulmonary hypertension in Colombia between 2022 and 2023.

Variable	*n* = 1045	%
Age (mean; SD)	62.9 ± 18.2	
Female gender	756	72.3
Age group (years)		
18 to 35	98	9.4
36 to 55	249	23.8
56 to 70	290	27.8
> 70	408	39
Region		
Bogota‐Cundinamarca	472	45.2
Central	217	20.8
Pacific	162	15.5
Caribbean	155	14.8
Oriental	37	3.5
Amazon	2	0.2
Prescribing specialty		
General practitioner	842	80.6
Neurology	52	5.0
Pneumology	41	3.9
Internal Medicine	22	2.1
Cardiology	21	2
Rheumatology	7	0.7
Others	60	5.7
Health system affiliation regime		
Contributory	889	85.1
Principal diagnosis ICD‐10^a^		
Primary pulmonary hypertension	1027	98.3
Chronic thromboembolic PH	18	1.7

Abbreviation: PH, pulmonary hypertension.

^a^ Main diagnosis using ICD‐10 codes, related to the inclusion criterion.

### 3.2 Patterns of Drug Use for the Treatment of Pulmonary Hypertension

A total of 947 (90.6%) patients received monotherapy, and 98 (9.4%) received combination therapy at the beginning of follow‐up. The most frequently used drugs for the treatment of this group of patients with PH were calcium channel blockers, particularly amlodipine and nifedipine, followed by phosphodiesterase 5 inhibitors such as sildenafil and tadalafil. Patients receiving endothelin receptor antagonists such as bosentan and macitentan and guanylate cyclase stimulants such as riociguat, among others, were also identified. The relationship between the mean dose and DDD showed that higher doses are being used for sildenafil, tadalafil, riociguat, amlodipine, treprostinil, and ambrisentan (compared with the dose defined by the World Health Organization for the main indication). Table 2 shows the patterns of use of this group of drugs, including frequencies of use, means and ranges of doses, dosing intervals, and age and sex of the patients for each drug.

**TABLE 2:** Patterns of use of drugs for pulmonary hypertension treatment in a group of 1045 patients in Colombia between 2022 and 2023.

Drug	*n* = 1045	%	Mean dose (mg/day)	nDDD^a^	Range, minimum and maximum dose mg/day	Most frequent dosing interval	Age (mean; SD)	Female proportion (%)
Calcium channel blockers								
Amlodipine	374	35.8	7.0 ± 2.6	1.4	5–20	QD	72.2 ± 13.1	69.2
Nifedipine	164	15.7	33.6 ± 9.8	1.1	20–60	QD	66.7 ± 14.9	84.1
Verapamil	32	3.1	171.4 ± 57.9	0.7	80–240	BID	75.4 ± 11.7	84.3
Diltiazem	26	2.5	93.3 ± 29.9	0.4	60–120	BID	69.1 ± 12.1	88.4
Levoamlodipine	11	1.8	3.0 ± 1.1	NA	2.5–5	QD	65.5 ± 13.0	72.7
Phosphodiesterase‐5 inhibitors								
Sildenafil	363	34.7	124.2 ± 42.3	2.5	60–300	TID	54.6 ± 18.8	72.1
Tadalafil	66	6.3	16.2 ± 6.5	1.6	5–20	QD	55.4 ± 17.4	51.5
Endothelin receptor antagonists								
Bosentan	160	15.3	206.1 ± 62.1	0.8	62.5–250	BID	51.2 ± 62.1	75.0
Macitentan	107	10.2	10.1 ± 0.8	1.0	10–20	QD	52.1 ± 19.1	71.9
Ambrisentan	73	7	10 ± 0.0	1.3	10–10	QD	53.4 ± 14.9	82.1
Soluble guanylate cyclase stimulators								
Riociguat	102	9.8	6.9 ± 1.2	1.5	1.5–7.5	TID	60.3 ± 16.2	62.7
Inhaled prostacyclin analogs								
Iloprost	19	1.8	15.9 ± 2.3	NA	15–30	6/day	50.7 ± 11.9	84.0
Prostacyclin analogs								
Treprostinil	12	1.1	5.9 ± 3.1	1.4	1–10	QD	47.0 ± 19.8	58.3
Prostacyclin receptor agonists								
Selexipag	9	0.9	1242 ± 516.4	0.6	400–2000	BID	45.0 ± 14.0	77.7

Abbreviation: SD, standard deviation.

^a^ Relationship between the average dose and the defined daily dose.

### 3.3 Treatment Schemes Received

**FIGURE 1:**
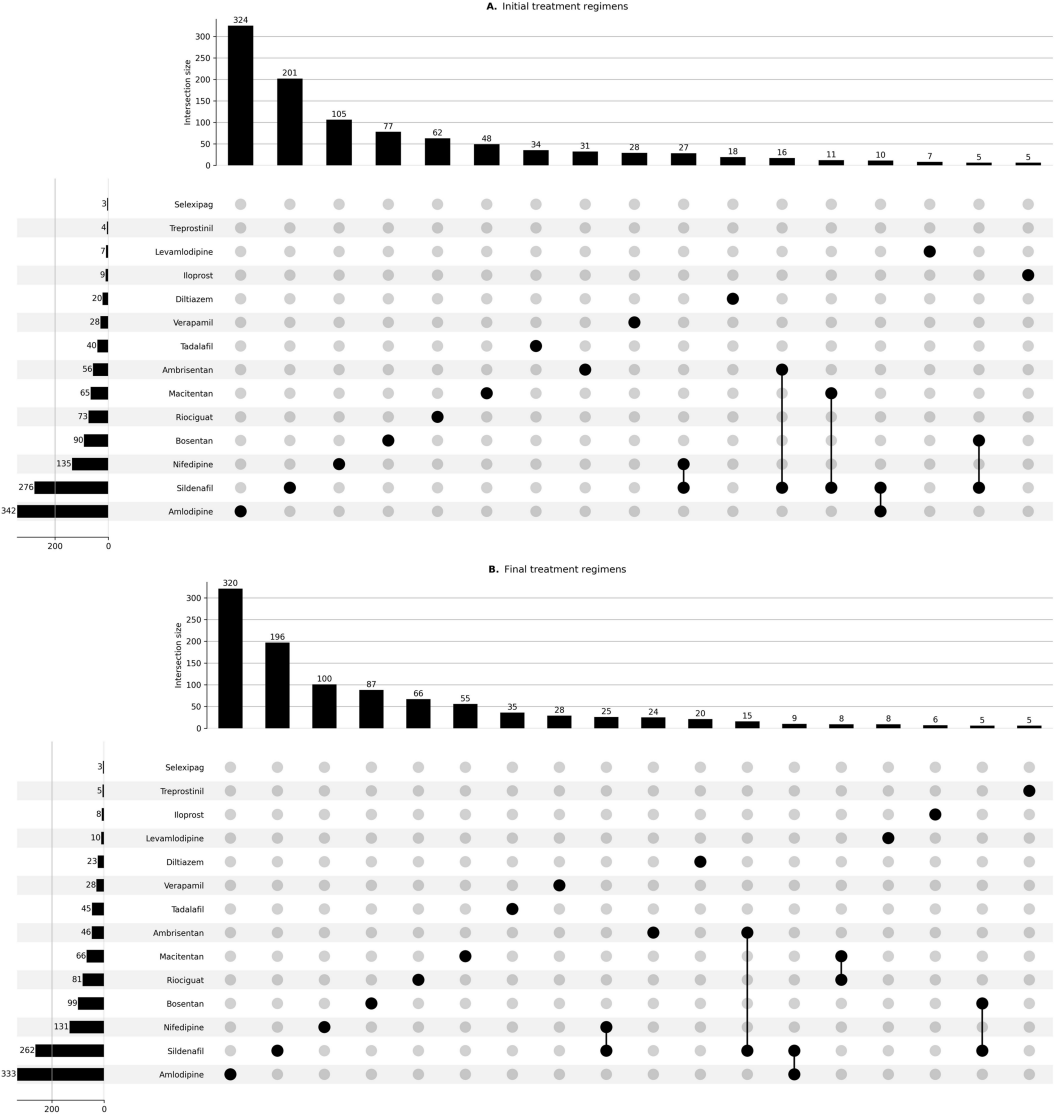
(A) Frequency of use of the main initial schemes of treatment of a group of patients with pulmonary arterial hypertension in Colombia, 2022–2023. (B) Frequency of use of the main final schemes of treatment of a group of patients with pulmonary arterial hypertension in Colombia, 2022–2023.

**FIGURE 2:**
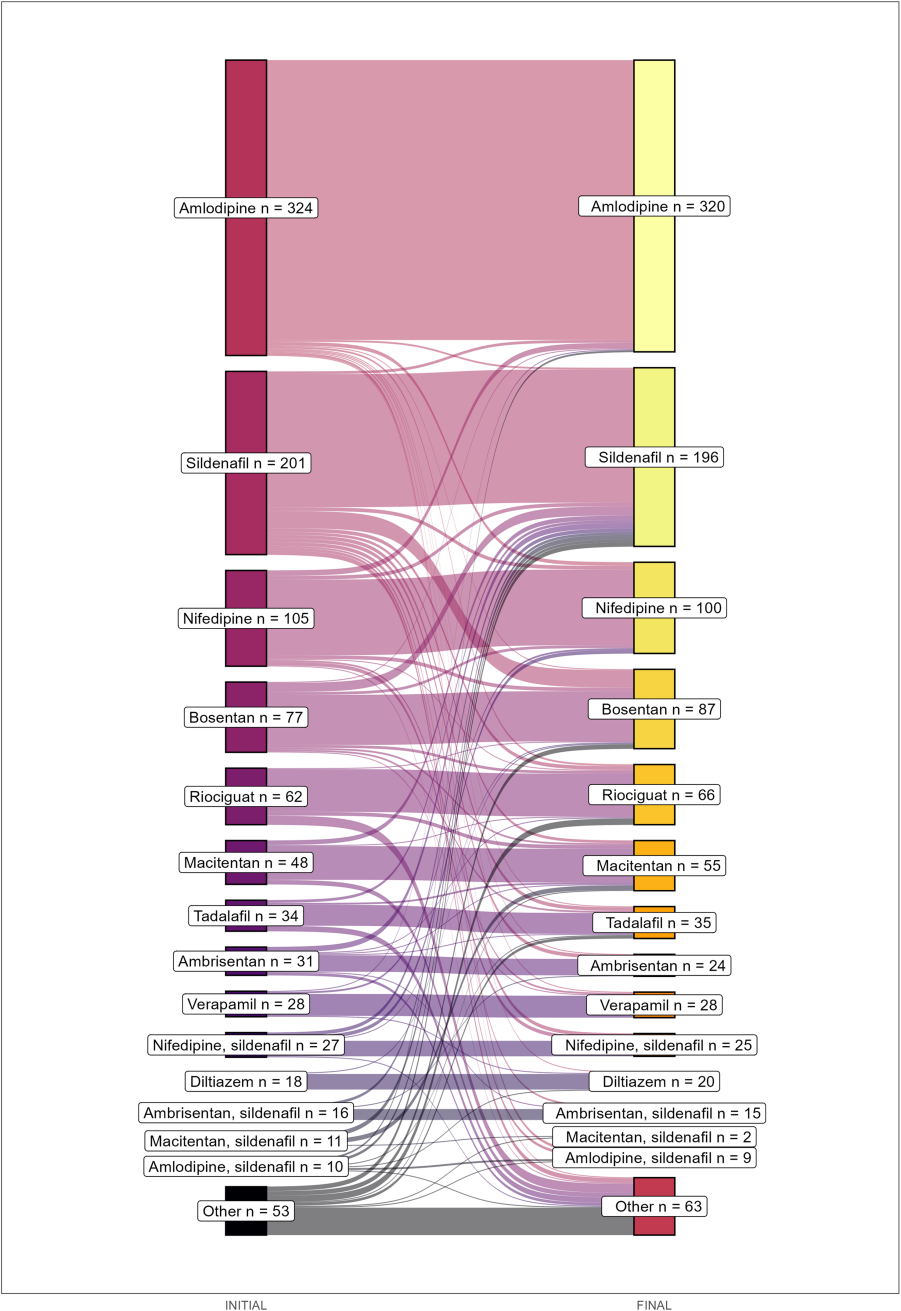
Sankey diagram with the main changes of the drugs used between the beginning and the end of the follow‐up of a group of patients with pulmonary arterial hypertension in Colombia, 2022–2023.

The treatment regimens that were used most frequently, both at the beginning and at the end of the follow‐up, were monotherapy with amlodipine, sildenafil, or nifedipine. On the other hand, the main combinations were sildenafil with ambrisentan or with macitentan and sildenafil with nifedipine, with similar behavior at the end of the follow‐up. Figure 1a,b shows the frequency of use of the main initial and final schemes of the patients evaluated. Figure 2 presents a Sankey diagram with the main changes of the drugs used between the beginning and the end of the follow‐up.

### 3.4 Persistence of Drug Use

Eighty‐nine patients (8.5%) had a single prescription of any of the drugs of interest, without any continuity in the treatment received. The persistence in general, for the group of patients who started with a single drug and were receiving monotherapy until they stopped, changed, or added a new drug, was 161 ± 123 days during 1 year of follow‐up. Persistence was higher than average for the group of patients who received calcium channel blockers and soluble guanylate cyclase stimulators (riociguat), whereas for phosphodiesterase 5 inhibitors, endothelin receptor antagonists and prostacyclin receptor agonist were less common. The group of patients treated with drug combinations (*n* = 98, 9.4%) had a mean persistence of 175 ± 127 days until the suspension or change of therapy, being the most frequent in combination with the endothelin receptor antagonists and soluble guanylate cyclase stimulators. The persistence identified for all patients undergoing treatment for PH was 163 ± 123 days. Table 3 shows the persistence of use for each of the drugs and their combinations, with their ranges and time percentiles.

**TABLE 3:** Persistence of use of drugs for pulmonary hypertension treatment in a group of 1045 patients with pulmonary hypertension in Colombia between 2022 and 2023.

Drugs	Mean	SD	Minimum	Maximum	Percentile		
					25th	50th	75th
Monotherapy							
Calcium channel blockers							
Levoamlodipine	206	131	30	379	103.5	220	303
Amlodipine	196.8	128.9	30	391	65.5	181.5	339
Verapamil	193.4	117.7	30	386	104	154.5	285
Diltiazem	189.1	114.9	30	363	109.5	167.5	301.8
Nifedipine	150.3	106.7	30	383	62	125	215
Soluble guanylate cyclase stimulators							
Riociguat	199.2	134.2	30	377	58.5	192	337.5
Phosphodiesterase‐5 inhibitors							
Sildenafil	136.7	115.9	30	382	30	92	205
Tadalafil	126.6	109.3	30	383	30	100	204
Endothelin receptor antagonists							
Ambrisentan	116.5	116.9	30	375	30	60	183.5
Macitentan	114.9	95.3	30	345	30	84.5	169.5
Bosentan	120	115.1	30	376	30	63	170
Prostacyclin receptor agonists							
Selexipag	111.7	141.5	30	275	30	30	152.5
Prostacyclin analogs							
Treprostinil	73	53.7	30	141	30	60.5	103.5
Inhaled prostacyclin analogs							
Iloprost	78.6	59.4	30	174	30	68	91
Main combinations							
Ambrisentan + Riociguat	259.3	340	175.5	380	199	340	360
Bosentan + Riociguat	187	192	103.5	305	136.3	192	242.8
Ambrisentan + Sildenafil	184.9	149	133.3	368	62.8	149	317.3
Macitentan + Sildenafil	182.7	127	139.3	384	78	127	325.5
Nifedipine + Sildenafil	172.9	119	135.5	387	45.5	119	312.5
Amlodipine + Sildenafil	153.2	148	103.5	348	93.5	148	178
Bosentan + Sildenafil	135	94	110.8	320	89	94	142

### 3.5 Comedications for Comorbidities

The most frequently used medications for other comorbidities were antihypertensives, especially angiotensin 2 receptor antagonists, beta‐blockers, and loop diuretics. More than half of the patients (53%) received statins, proton pump inhibitors, and acetaminophen. Table 4 shows the frequencies and proportions of the concomitant medications prescribed.

**TABLE 4:** Concomitant medications received by a group of 1045 patients with pulmonary hypertension in Colombia between 2022 and 2023.

Concomitant medications	(*n* = 1045)	%
Antihypertensives		
Angiotensin receptor blockers (ARB)	493	47.2
Beta‐blockers	371	35.5
Loop diuretics	339	32.4
Mineralocorticoid receptor antagonists	228	21.8
Thiazides	153	14.6
Angiotensin converting enzyme inhibitor (ACEi)	116	11.1
Alpha 1 and central blockers	62	6
Neprilysin receptor antagonist (ARNI)	33	3.2
Lipid‐lowering drugs		
Statins	523	50
Fibrates	36	3.4
Ezetimibe	3	0.3
Others for cardiovascular use		
Antiarrhythmics	20	1.9
Hematic system and others		
Acetylsalicylic acid	336	32.2
Direct oral anticoagulants	193	18.5
Warfarin	64	6.4
Clopidogrel and other antiplatelet agents P2Y12i	42	4
Antihistamines		
2nd generation antihistamines	136	13.0
1st generation antihistamines	117	11.2
Levothyroxine	289	27.7
Digestive		
Proton pump inhibitors	563	53.9
Analgesic and anti‐inflammatories		
Acetaminophen	555	53.1
Nonsteroidal anti‐inflammatory drugs (NSAIDs)	248	23.7
Systemic corticosteroids	180	17.2
Partial agonist opioid	170	16.3
Full agonist opioid	74	7.1
Dipyrone	51	4.9
Respiratory		
B2 agonists	168	16.1
Inhaled anticholinergics	227	21.7
Psychotropic drugs		
Anticonvulsants	221	21.1
Selective serotonin reuptake inhibitors	123	11.8
Trazodone	69	6.6
Atypical antipsychotics	52	5.0
Tricyclics antidepressant	35	3.3
Benzodiazepines	28	2.7
Selective serotonin + norepinephrine reuptake inhibitors	21	2.0
Z drugs	16	1.5
Conventional antipsychotics	13	1.2

## 4 Discussion

With the present study, it was possible to establish the treatment patterns of patients with PH in Groups 1 and 4 from a database of drug claims. This study provides the first comprehensive description of its characteristics, the drugs used, the combinations and the persistence of use during 12 months of follow‐up. These findings are consistent with sociodemographic data from epidemiological studies of PH published worldwide, where a female predominance and an average age of 62 years were reported [8, 9], and according to the cluster identified by IPAH in the COMPERA registry [10]. However, the diagnosis is usually made between the ages of 30 and 60, according to international reports [11].

More than 90% of the patients received monotherapy, although different reports from clinical trials and meta‐analyses have shown that combined therapy is more effective, offering up to a 35% reduction in clinical worsening [12]. This fact is usually determined by the 6‐min walking test [13] according to the recommendation of the Colombian Consensus on Pulmonary Hypertension [5]. In addition, compared with the benefits of combined therapy, the meta‐analysis published by Lajoie et al. reported that the use of combined therapy, in addition to significantly improving the results of the 6‐min walking test, has a favorable effect on reducing hospitalizations and the need for treatment escalation and leads to a reduction in symptomatic progression [12]. In this way, a high proportion of Colombian patients could benefit from more intensive therapy that achieves greater benefits and avoids the complications and increased costs derived from the decompensation of their disease [14].

The most frequently used drug is amlodipine, a calcium channel blocker widely used in the pharmacological therapy of patients with PH, especially those who present a positive acute vasoreactivity test, which can achieve an adequate therapeutic response [2, 15]. However, the high frequency of use of this therapy is striking owing to its restrictions on its use in this diagnosis given that, according to the 2022 European ESC/ERS Guidelines on Pulmonary Hypertension, the use calcium channel blockers is not recommended if the vasoreactivity test is negative. In this case, its use could be possibly explained to a concurrent medication for a cardiovascular‐type comorbidity [2]. Likewise, this could occur for different reasons, including a lack of knowledge of the current therapeutic recommendations, difficulties in accessing the vasoreactivity test, loss to follow‐up by the pulmonology specialist, or limited access to specific medications for the disease.

On the other hand, the second most commonly used drug was sildenafil. Even though the recommended dose is 20 mg three times a day (60 mg/day) [2, 16], the average dose observed in this analysis was more than double of the suggested by the clinical practice guidelines, with the dosage range even reaching 300 mg/day. This translates to a DDD of 2.5 higher being used, putting patients at an increased risk of severe adverse reactions such as hypotension and severe vasodilation [17].

The treatment persistence in these patients during the year of follow‐up was 161 days, which is striking for several reasons, including (a) that a significant number of patients (8.5%) had a single prescription, although the reasons for its suspension or lack of continuity are unknown, taking into account that medications for PH should not be suspended suddenly [18], (b) lack of adherence by the patient, as several medications are dosed three or more times per day, (c) administrative difficulties for access to therapy, and (d) changes in diagnosis, which may occur after confirmation of the diagnosis with right heart catheterization performed after the echocardiogram and can cause changes in the treatment schemes [19].

Because this is the first assessment of the persistence of drug use in pulmonary arterial hypertension in Colombia and Latin America, there are no previous reports with which to compare similar populations; however, notably, the Colombian Consensus on Pulmonary Arterial Hypertension recommends a minimum paraclinical follow‐up every 6 months, which is why the prescribed therapy can be changed with the aim of improving tolerability, achieving symptomatic control and decreasing the frequency of complications [5].

The drug with the longest persistence of use was riociguat (mean of 199.2 days), which is recommended to start with a titration phase with a dose of 1.0 mg three times a day and a progressive increase of 0.5 mg every 2 weeks up to a maximum dose of 2.5 mg three times a day as tolerated, which is surely associated with a higher follow‐up time for the patient, allowing evaluation of the drug's effectiveness and tolerability [20]. Among the co‐medications that patients received, the significant use of antihypertensives such as renin‐angiotensin‐aldosterone system blockers and beta‐blockers, as well as other cardiovascular drugs, is striking, showing a significant frequency of this type of comorbidity, similar to that identified in patients with IPAH in the COMPERA registry [10], and also the use of anticonvulsants in 21% of the population evaluated, although the indication was not identified, these medications are widely used not only in epilepsy but also in indications such as bipolar affective disorder, neuropathic pain, migraine, or insomnia [21, 22].

Riociguat is a good option in patients for whom the therapeutic goals with phosphodiesterase 5 inhibitors are not achieved [23, 24]. Early transition has been shown to be associated with improvements in the clinical parameters of the disease; in addition, early transition is well tolerated. In fact, current treatment guidelines recommend the transition from PDE5i to riociguat in patients with an intermediate‐low risk of mortality (class IIb recommendation) [25, 26]. In the present study, it was observed that the combination with the greatest persistence was riociguat + ambrisentan; this therapy is reported to be highly effective in reducing pulmonary vascular resistance, although it can be associated with severe adverse reactions [2, 24].

Among the limitations of this study, it is important to consider that the source of information was a database of drug claims, in which each patient was identified according to the diagnosis recorded per the ICD‐10, which be, in some cases, inaccurate. In addition, owing to the very nature of the study, it was impossible to obtain paraclinical diagnostic and follow‐up data, including 6‐min walking test data that support the severity of the pathology and the relevance of each of the prescriptions used in the patients. As this is a study of prevalent patients with PH, it was not possible to establish the age of the patients at the time of diagnosis. Likewise, owing to the low number of patients identified with CTEPH, it was not possible to perform an individualized analysis for each PH group.

The findings of this study can be extrapolated to populations with insurance and drug access characteristics like those of the general Colombian population. Most of the prescriptions were made by general practitioners despite the disease being a highly complex pathology that requires management by specialists in pulmonology; therefore, it is a call to attention for most countries, not only for Colombia but also for those in the process of development to address this pathology with greater interest. The strength of this study is its large population and monitoring of the persistence of pharmacological therapy, which has not been previously not described in the region.

## 5 Conclusions

Based on the above findings, it can be concluded that PH in Colombia is treated predominantly with monotherapy, calcium channel blockers, and phosphodiesterase 5 inhibitors. However, current clinical practice guidelines recommend the use of combined therapy, which could not be observed in this study. The average persistence of the use of drugs for treatment for less than 6 months may be associated with difficulties in follow‐up, adherence, effectiveness, tolerability, and access, which should be investigated and, if possible, resolved. These results underscore the need to improve continuous medical education processes and highlight the current therapeutic difficulties of patients diagnosed with PH.

## Author Contributions

M.M.D.: wrote manuscript, designed research, performed research, analyzed data. A.G.M.: analyzed data, wrote manuscript. L.F.V.R.: analyzed data. M.P.: designed research, analyzed data. J.S.F.: designed research, analyzed data. M.d.R.F.: designed research, analyzed data. R.S.: designed research, analyzed data. O.P.: designed research, analyzed data. J.E.M.A.: wrote manuscript, designed research, performed research.

## Disclosure

In no case were artificial intelligence tools used for the construction, data analysis, or writing of the final manuscript.

## Ethics Statement

The study protocol was approved by the bioethics committee of the Universidad Tecnológica de Pereira, Colombia (approval code: 128‐201123). According to Resolution 8430 of the Colombian Ministry of Health, observational research carried out on clinical records does not require informed consent.

## Consent

Consent to participate is not applicable as this was a retrospective observational study. All authors consent to publish this study.

## Conflicts of Interest

Juan‐Sebastian Franco, Maria del Rosario Forero, Rubiela Suares, and Oscar Penuela are full‐time employees of Bayer Colombia. Manuel Machado‐Duque, Andres Gaviria‐Mendoza, Luis Fernando Valladales‐Restrepo, and Jorge E. Machado‐Alba have a contractual relationship with Audifarma SA. Manuel Pacheco is a professor of Universidad Tecnológica de Pereira.

## Data Availability

The data that support the findings of this study are available from DOI: dx.doi.org/10.17504/protocols.io.rm7vzjok8lx1/v1 (private link for reviewers: https://www.protocols.io/private/1B61C5EC486411EF875E0A58A9FEAC02 to be removed before publication.)
